# Propranolol versus Other Selected Drugs in the Treatment of Various Types of Anxiety or Stress, with Particular Reference to Stage Fright and Post-Traumatic Stress Disorder

**DOI:** 10.3390/ijms231710099

**Published:** 2022-09-03

**Authors:** Łukasz Szeleszczuk, Dawid Frączkowski

**Affiliations:** Department of Physical Chemistry, Chair and Department of Physical Pharmacy and Bioanalysis, Faculty of Pharmacy, Medical University of Warsaw, Banacha 1 Street, 02-093 Warsaw, Poland

**Keywords:** propranolol, anxiety, stage fright, PTSD, post-traumatic stress disorder

## Abstract

Propranolol, a non-cardioselective β_1,2_ blocker, is most commonly recognised for its application in the therapy of various cardiovascular conditions, such as hypertension, coronary artery disease, and tachyarrhythmias. However, due to its ability to cross the blood–brain barrier and affinity towards multiple macromolecules, not only adrenoreceptors, it has also found application in other fields. For example, it is one of the very few medications successfully applied in the treatment of stage fright. This review focuses on the application of propranolol in the treatment of various types of anxiety and stress, with particular reference to stage fright and post-traumatic stress disorder (PTSD). Both mechanisms of action as well as comparison with other therapies are presented. As those indications for propranolol are, in most countries, considered off-label, this review aims to gather information that can be useful while making a decision about the choice of propranolol as a drug in the treatment of those mental conditions.

## 1. Introduction

**Figure 1:**
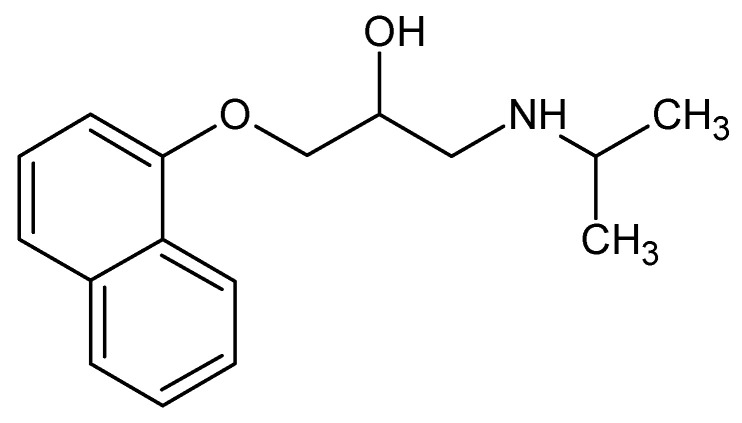
Chemical structure of propranolol.

**Figure 2:**
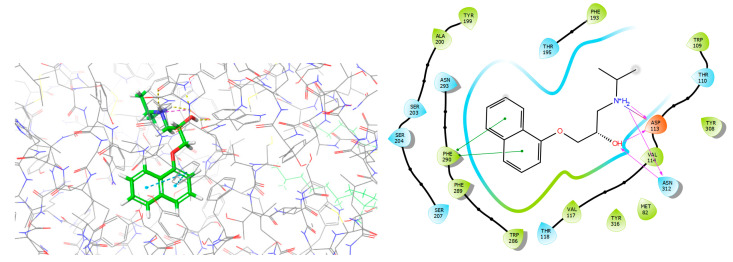
Complex of β_2_-adrenergic receptor with propranolol, structure from RCSB PDB, ref. code 6PS5.

Propranolol (Figure 1), discovered by Nobel Prize laurate Sir James Black, was patented in 1962 and approved for medical use in 1964 [1]. It is classified as a competitive non-cardioselective sympatholytic beta blocker that crosses the blood–brain barrier and, thus, exerts effects on the central nervous system, in addition to its peripheral activity [2]. Besides its ability to block adrenergic receptors (Figure 2), propranolol is a weak indirect α_1_-adrenoceptor agonist [3]. There is also evidence that this API may act as a weak antagonist of certain serotonin receptors, namely, 5-HT_1A_, 5-HT_1B_, and 5-HT_2B_. This broad biological activity of propranolol explains why, soon after its discovery in the early 1960s, Turner and Granville-Grossman observed its anxiolytic effects [4]. Since then, propranolol has gained increasing interest in the field of psychiatry, among others, for the treatment of various types of anxiety and stress. This review focuses on the application of propranolol in the therapy of anxiety disorders, with particular emphasis on stage fright and post-traumatic stress disorder (PTSD).

The aim of this general review is to present information on both the molecular basis as well as clinical studies describing the application of propranolol in the treatment of PTSD and stage fright. As those conditions are very complex, with symptoms not limited solely to those of the central nervous system (CNS) but also including somatic symptoms, it is important to understand the mechanism of action of propranolol with respect to those psychiatric conditions, especially since new molecular targets of propranolol have been discovered in recent years.

## 2. Anatomical Therapeutic Chemical (ATC) Classification System

Propranolol (ATC code: C07AA05) has been classified to the pharmacological subgroup C07A (beta blocking agents) in the main anatomical group C, which are drugs for the cardiovascular system. There are many drugs within the pharmacological subgroup C07A, which have been classified according to their mechanism of action.

There are 3 subgroups:

- C07AA—beta blocking agents, non-selective;
- C07AB—beta blocking agents, selective;
- C07AG—alpha and beta blocking agents.

Propranolol belongs to the C07AA subgroup, which also includes: alprenolol, oxprenolol, pindolol, timolol, sotalol, nadolol, mepindolol, carteolol, tertatolol, bopindolol, bupranolol, penbutolol, cloranolol, and carazolol [5].

## 3. Stage Fright

Stage fright (also referred to as performance anxiety) is a group of disorders affecting individuals in a range of endeavours, such as public speaking, sport, and the performing arts in dancing, acting, and music making. Typical symptoms of stage fright are similar to those occurring in other types of anxieties. They include negative mood, typically accompanied by bodily symptoms such as increased heart rate (HR), muscle tension, trembling, hyperventilation, nausea, dry mouth, shaky voice, blushing, sweating, light-headedness, and a sense of unease and apprehension about the future [6].

Stage fright is a serious problem for many professional and amateur artists, as severe difficulties resulting from performance anxiety may lead to premature termination of promising careers [7,8]. Among performing artists, instrumentalists seem to be the most affected by anxiety, followed by singers, dancers, and actors [9]. Importantly, stage fright is not limited to the period immediately before and during performance. It can occur days, weeks, or even months before the show [10]. For all these reasons, performance anxiety represents a considerable health and social problem.

## 4. Post-Traumatic Stress Disorder (PTSD)

Individuals who are exposed to life-threatening trauma, such as wartime combat, assault, rape, car accidents, natural disasters, man-made traumas, or worldwide pandemics [11], are at risk of developing PTSD. People with PTSD suffer from severe psychological distress as a result of having to relive their trauma repetitively through intrusive flashback memories [12,13,14]. Other severe symptoms accompany these memories, such as emotional numbing, avoidance of trauma-related stimuli, and a constant state of increased arousal and hypervigilance [15,16,17]. Cognitive dysfunction has been identified in PTSD and linked to impaired traumatic memory processing [18], elevated psychiatric symptom severity, and functional disability [19]. The ability of the hippocampus (HPC, the main location for memory storage) to control the memory of traumatic events is also found to be dysfunctional [20]. Anxiety and sleep difficulties are some of the debilitating and persistent symptoms of PTSD [21]. PTSD patients usually have heightened resistance to extinction learning [22]. This disorder has been linked to an increase in the frequency of suicide behaviours, such as suicidal thoughts, plans, or actions, as well as other mood disorders and anxiety symptoms [23,24]. According to paediatric studies, children with burns who do not receive adequate pain management may develop PTSD [25,26]. Children with PTSD often exhibit avoidance behaviours, increased arousal, flashbacks, and nightmares [27].

In its fifth edition of its Diagnostic and Statistical Manual of Mental Disorders (DSM-5), the American Psychiatric Association updated the PTSD diagnostic criteria in 2013 [28]. The DSM-5 diagnostic criteria list exposure to actual or threatened death, significant injury, or sexual abuse as the numerous specific triggers for PTSD. Regardless of its cause, the ensuing disturbance results in clinically substantial distress or a deficiency in the person’s capacity for social interaction, employment, or other critical areas of functioning. DSM-5 merely requires that the disturbance persist for longer than a month and eliminates the distinction between acute and chronic phases of PTSD [29]. However, at least a third of those who initially develop PTSD continue to face symptoms for three years or longer [21].

PTSD affects about 8% of the population in the United States [30,31]. Females are more than twice as likely as males to develop PTSD following a traumatic event [32]. The Food and Drug Administration (FDA) has approved only two medications to treat it (paroxetine hydrochloride and sertraline hydrochloride), both with limited efficacy [30,31]. Trauma-focused psychotherapies enable PTSD remission. The advantages of such treatments decline over time [33].

The hypernoradrenergic state is associated with the pathophysiology of PTSD [34]. Noradrenaline (norepinephrine, NE) and adrenaline, which cause stress, are often found in higher concentrations in PTSD patients [29]. It is believed that adrenaline helps memory consolidation and contributes to the reoccurrence of PTSD symptoms [35]. Given the high and positive correlation between cerebrospinal fluid (CSF) NE levels and the severity of PTSD symptoms [34], one possible explanation is that traumatic memory reactivation in a patient with severe PTSD may result in the release of a higher level of NE than in a patient with non-severe PTSD. Furthermore, PTSD patients’ HR or blood pressure (BP) have increased in reaction to stressful signals, demonstrating conditioned fear responses [36,37]. The discovery that memories were pliable and changeable rather than fixed triggered a series of studies that totally altered how PTSD is treated today [20].

## 5. The Medical Use of Propranolol

Propranolol, being a non-specific β_1_- and β_2_-adrenergic receptor antagonist, was initially prescribed for cardiac disorders, which has remained its main clinical prescription. However, during the years of consecutive studies and clinical trials, more indications have been added to its characteristic. Those include treatment of conditions from various fields, such as psychiatric, ophthalmological, hormonal, and even neoplastic [1].

### 5.1. Cardiovascular

Although propranolol was first developed to treat angina pectoris, its use in other cardiovascular conditions, such as hypertension, cardiac arrhythmias, and myocardial infarction, was soon recognised [38,39,40,41].

Despite many new generation cardioselective beta-blockers being now available, propranolol continues to be used in specific conditions. For example, propranolol has been found to be as effective as carvedilol on left ventricular volume and function after primary coronary stenting in acute myocardial infarction [42]. Barton et al. assessed the efficacy and safety of high-dose propranolol for the management of supraventricular tachyarrhythmias (SVTs) in infants (N = 287) [43]. Bonten et al., in a meta-analysis including 31 studies, found that propranolol significantly decreased platelet aggregation [44]. Propranolol has also been extensively used for the management of essential hypertension [45].

### 5.2. Psychiatric

According to a meta-analysis, propranolol may be useful in the treatment of anxiety disorders caused by unsettling memories, notably PTSD [46]. Furthermore, propranolol is useful in lowering emotional arousal [47], eradicating stage fright [48], and alleviating anxiety-related cognitive dysfunction [49].

#### 5.2.1. Stress

It is important to consider propranolol’s potential as a preventative treatment for chronically stressed individuals [50]. Propranolol reduced anxiety-like behaviours while also increasing resilience to a following stressor [51]. An oral dose of 80 mg propranolol lowered the rise in HR and systolic BP brought on by stress to 49.9% and 8.3%, respectively, as opposed to 61.0% and 17.4% with placebo. Additionally, propranolol markedly reduced the skin’s temperature increase on the trunk [52].

##### PTSD

Propranolol has also shown promise for the treatment of chronic PTSD [53]. This β-AR antagonist was found to block memory reconsolidation in healthy humans in a fear conditioning test [54], and studies have reported successful propranolol-induced reconsolidation disruption that lasted at least one month and was resistant to fear reinstatement [46]. Fear memory reconsolidation is the process by which reactivation by exposure to the conditioned stimulus (CS) or an unconditioned stimulus (US) makes memory traces labile, thereby triggering transient protein destabilisation that can be modified by pharmacological and behavioural interventions for several hours after memory reactivation [55]. Fear memories can be updated with new information thanks to the reconsolidation process [55].

The first clinical trial in PTSD patients was conducted by Brunet et al. [56]. In this study, it was discovered that providing chronic PTSD patients (N = 19) 60 mg of long-acting oral propranolol followed by 40 mg of short-acting oral propranolol significantly decreased physiologic response to the memory one week later. Oral propranolol given 1 h before retrieval of the US reduced subsequent fear responses and disrupted connections between all CSs and the US [57]. The effects of propranolol administered one hour before exposure to emotionally charged or neutral storylines were investigated by Cahill et al. [58]. In comparison to the control group, participants who received propranolol remembered fewer emotional details. Propranolol administered within the first 6 h after a traumatic event significantly reduces the likelihood of developing PTSD [59,60]. This drug appeared to be effective during the reconsolidation time window, which lasted approximately 6 h and enabled the original memory to be updated with new protein synthesis [61,62,63]. The physiological reactivity and HR of those who received propranolol 4–12 h after the trauma reportedly improved [64]. Moreover, administering propranolol three times per day for a week after a trauma minimised the onset of PTSD symptoms [59]. In a randomised clinical trial [65] with “pre-reactivation propranolol therapy”, PTSD participants (N = 60) who actively recalled their traumatic event under the influence of propranolol once a week for up to six weeks showed a significant decrease in symptom scores (PTSD symptom improvement = 36%) compared to those who received pre-reactivation with the placebo (PTSD symptom improvement = 13%). Chronic propranolol treatment may be more beneficial in alleviating PTSD symptoms, according to certain human studies [46,60]. Additionally, in patients with severe PTSD symptoms (the PTSD Check List ≥ 65; PCL-S ≥ 65) before treatment, PCL-S and the Beck Depression Inventory-II (BDI-II) scores continued to decline three months after the end of treatment in the propranolol group, while they increased in the placebo group [66]. Brunet et al. discovered that the physiological response to trauma reactivation was low even at four months following the therapy [67]. Although this therapy has been referred to as "forgetting therapy", its goal is to help patients separate their emotions and worries from their memories rather than making them forget their physical experiences. Even five months after starting to take propranolol, the advantage was still evident: the flashbacks completely stopped occurring, and the emotional reaction to the nightmares was greatly reduced. In a case report, it has been described that the treatment with propranolol resulted in a decrease in alcohol use and an improvement in the patient’s quality of life [29].

Following memory reactivation, this API can decrease the identification of emotional pictures, according to additional research utilising functional magnetic resonance imaging [68]. A propranolol-mediated decrease in sympathetic tone in the period surrounding re-exposure can lead to changes in the subjective qualities of emotional memories, which is compatible with propranolol’s anxiolytic effects [48,67,69,70,71]. A meta-analysis of healthy humans found that a single memory reactivation session combined with propranolol medication lowered the strength of negatively valenced emotional memories [46]. Propranolol can disrupt a fear memory that is specific to the reactivated CS when administered during CS retrieval-induced reconsolidation [57]. Furthermore, the propranolol-induced suppression of fear memory reconsolidation retrieved by the US was effective for remote fear memory and exhibited a reasonably long-lasting effect (at least two weeks) [57]. In humans, the unconditioned stimulus-based memory retrieval interference procedure with propranolol can permanently reduce the fear response and prevent the return of fear for all CSs [57]. Numerous follow-up studies of propranolol administration after reactivation in humans convincingly confirmed attenuated emotional responses while also demonstrating that it preserved declarative memory, i.e., knowledge of CS–US contingencies remained unaffected [72,73,74,75,76,77,78]. A case series found that providing 40 mg of propranolol shortly after reactivation decreased PTSD symptoms [79]. Six brief trauma reactivation sessions under the influence of propranolol resulted in considerable PTSD symptom improvement in three other independent open-label studies. Patients who all developed PTSD as a result of the industrial disaster in Toulouse in 2001 were compared; 8% in the control group lost their diagnosis, compared to 86% of treated patients [69].

Propranolol therapy seems to be a potentially safe and effective therapeutic option for children with PTSD symptoms [80]. In a pilot trial, Famularo et al. [81] investigated the advantages of propranolol therapy for children with PTSD who had suffered severe physical and sexual abuse. While using propranolol, children reported a considerable improvement in their PTSD symptoms.

Moreover, propranolol may alter visuospatial processes associated with the resolution of traumatic intrusions in those with chronic PTSD [53]. After taking propranolol, HR drops were associated with improved Perceptual Organisation (PO) performance, which may point to peripheral and central β-adrenergic blockade effects on cognition, specifically during visuospatial processing and visual attention in PTSD [82]. Additionally, propranolol decreased diastolic BP, which was associated with less severe PTSD symptoms [53]. In addition, propranolol may improve Processing Speed (PS) performance in PTSD patients as compared to placebo [53]. In combination with extinction training and when administered soon after trauma, propranolol may be most effective at reducing long-term fear [83].

#### 5.2.2. Anxiety

The majority of mental health disorders are anxiety-related [84], and they have significant psychological, social, and financial implications [85]. A common anxiolytic drug with minimal effects on cognition is propranolol hydrochloride [2,86]. The use of propranolol to mask the physical symptoms of anxiety is well established [29]. This medicine reduces the physiological symptoms of anxiety, such as BP, changes in HR, respiration rate, and skin conductance [2]. According to Granville-Grossman and Turner’s [87] cross-over study, patients with anxiety states were evaluated to be significantly better after two weeks of propranolol treatment compared to two weeks of placebo. Patients with chronic anxiety were treated with propranolol at a dose of 160 mg daily. After two weeks of treatment, propranolol severely decreased the physical aspects of anxiety, and it also significantly relieved other symptoms including shortness of breath, chest pains, and weakness that had some association to beta stimulation [88]. Experimental findings suggest that administering propranolol soon after retrieving an emotional memory can cause post-reactivation amnesia, which is the attenuation of the memory’s later expression [89]. It has been demonstrated that propranolol has an acute effect on fear-potentiated startle responses [89]. Additionally, studies on propranolol have indicated that it can alleviate less stressful conditions such as exam anxiety [90,91,92], stage fright [48], performance anxiety in musicians [93], and fear of surgery [94,95].

##### Stage Fright

For decades, beta blockers such as propranolol have been used to treat situational anxiety such as stage fright and exam- or interview-related anxiety [48,91]. Propranolol has been used in the clinic for its short-term effect in performance anxiety, including test taking and oral presentations [2]. Stage fear no longer causes any physical performance barriers thanks to beta blockade, which also results in the removal of the common dry mouth [48]. Considering that physical anxiety symptoms usually worsen the patient’s anguish, providing the patient 40 mg of propranolol 1 h before a performance may be quite helpful [96]. According to experienced music critics, the level of musical performance substantially improves after taking this medication [48].

##### Social Anxiety Disorder (SAD)

The strategy for reducing anxiety during exposure treatment is to target psychophysiological arousal with propranolol administration, which makes anxiety during exposure more tolerable and may increase the chances of effectively coping with the feared situation. In patients with SAD, propranolol was combined with one session of exposure [97]. On the second day, there was a decrease in anxiety.

##### Fear of Public Speaking

Fear of public speaking is a ‘performance only’ subtype of SAD, characterised by extreme fear in, and avoidance of, public speaking situations, without more general social impairment as a result of anxiety [98]. It is one of the most widespread fears, and it can lead to missed educational, social, and professional opportunities [99]. Administration of propranolol during reconsolidation mitigated public speaking anxiety [98]. Regardless of participant body mass, 40 mg of propranolol was found to be an effective dose for fear neutralisation [100], and the medicine may be administered up to 1 h after reactivation [78].

#### 5.2.3. Phobias

Propranolol has been demonstrated to be effective in reducing anxiety in patients with dental phobia [101] and arachnophobia [74].

##### Fear of Dental Extraction

It has been shown that extractions raise the risk of developing chronic anxiety for dental surgical operations, acute types of dental anxiety (i.e., dental phobia), and post-traumatic stress symptoms [102]. One month after having their wisdom teeth surgically removed, a small percentage of patients, between four and eight percent, experience heightened dental trait anxiety or even PTSD symptoms [103,104]. Propranolol may be used to treat dental trait anxiety because traumatic memories appear to be crucial in its maintenance and severity [105]. Propranolol has a beneficial impact on dental anxiety and lessens the storage of fear memories. Furthermore, propranolol has the capacity to inhibit ‘memory reconsolidation’ (that is, it blocks the process of storing a recently retrieved fear memory) [102]. It has been observed that, as compared to placebo, 80 to 120 mg of oral propranolol significantly reduced self-reported states of anxiety during injection of a local dental anaesthetic [101].

##### Arachnophobia

Soeter and Kindt [74] used a live tarantula to reactivate a naturalistic fear memory in subjects who were afraid of spiders. This ‘memory reactivation’ was promptly followed by oral propranolol administration. Participants who received propranolol + reactivation showed significant reductions in their fear of spiders, and were able to touch or even hold spiders for at least a year after the intervention. On the other hand, those who received placebo + reactivation or propranolol alone showed no alterations in their fear. The fear levels of control participants remained stable, while reactivation + propranolol participants achieved immediate and significant reductions in their fear of spiders. These control conditions show that the fear reduction cannot be explained by propranolol’s general fear-dampening effect or by simply being exposed.

In addition, the eye-blink startle reflex was used to test the reaction to a loud noise combined with exposure to a fear-relevant stimulus (for example, photographs of spiders). Twenty-four hours after the drug’s administration, the behavioural reaction to the fear memory was completely erased. Furthermore, retrieval methods failed to reactivate the fear response [54,71,106].

However, 36 arachnophobic people participated in a double-blind, placebo-controlled trial conducted by Elsey and Kindt [107], who also used a reactivation procedure. They discovered a trend for better results in the placebo group, who showed larger improvement in phobic behaviour scores than the propranolol group [107].

#### 5.2.4. Autism Spectrum Disorder (ASD)

Propranolol may be beneficial to people who suffer from emotional, behavioural, and autonomic dysregulation (EBAD). This medicine also may help children with ASD and EBAD improve their therapy outcomes by alleviating symptoms related to autonomic dysregulation and/or hyperarousal. A considerable reduction in aggression, organic brain dysfunction [108,109,110], and anxiety disorders [111,112] has been reported in adolescents with ASD [113]. Moreover, propranolol was found to be effective in reducing self-injurious behaviours (SIBs) in people with ASD [114]. There is also some evidence that the use of propranolol may result in significant improvements in EBAD, the symptomatology of ASD, with a focus on cognitive performance and neural correlates and the management of behaviour, predominantly for aggression and SIBs [115]. Propranolol’s anxiolytic impact via the autonomic nervous system may be beneficial in people with greater physiological anxiety [116].

Individuals with ASD showed improvements in verbal problem solving [117,118], semantic processing [119], and working memory (WM) when administered propranolol [120]. Moreover, propranolol was found to improve functional connection in people with ASD [121,122]. These findings also point to propranolol’s ability to support cognitive processing. Greater associative processing and subnetwork integration may be obtained by regulating NE. As a result, people with ASD may have improvements in attention-shifting, sensory processing, language communication, and social information processing [121]. In addition, increases in nonverbal communication [123] and reductions in hypersexual behaviours [124] were noted. These benefits were found in studies that used a 40 mg propranolol dosage, with only one study utilizing a low dose of 20 mg [124]. Propranolol may be a potential therapy choice for patients with ASD who have complex symptoms [115].

#### 5.2.5. Other Psychoses

When benzodiazepines, anticonvulsants, and major tranquillizers failed to manage adult patients’ explosive rage outbursts and episodic belligerence, propranolol was used successfully [108]. The use of this medicine has been documented in case reports for the treatment of agitation in organic patients [125], aggressive behaviour following acute brain damage [126], and postencephalitic psychosis [127]. According to reports, the medication is also effective in treating acute postpartum psychosis [128]. Propranolol administered in 30–38 mg doses daily was found to significantly reduce uncontrollable rage outbursts in a study of 30 children and adolescents with organic brain dysfunction [110].

### 5.3. Other Uses

Other than those described above, therapeutic applications of propranolol include, but are not limited to, migraine prophylaxis [129], cluster headache prevention [130], and pheochromocytoma [131]. Propranolol has been also used in the treatment of various rare vascular diseases, including hereditary haemorrhagic telangiectasia, von Hippel–Lindau disease, paraganglioma syndrome, cerebral cavernous malformations, angiosarcoma, and tuberous sclerosis [132]. Oral propranolol administration seems to be an effective method to minimise the development of sight-threatening choroidal effusion after glaucoma surgery [133] as well as an effective and safe medication in the treatment of primary hyperhidrosis [134].

## 6. Mechanism of Action of Propranolol in the Treatment of Stage Fright

Beta-blocking medications used to treat stage fright act peripherally rather than centrally. The majority of the anxiolytic effect is produced by small doses of these drugs, since they are competitive inhibitors [48]. Many of the symptoms of performance anxiety, including tremor and palpitations, are brought on by an increase in the release of adrenaline and NE from the sympathetic nervous system and adrenal medulla, and medications that block beta-adrenoceptors, such as propranolol, minimise or eliminate these symptoms [135].

When beta blockers were administered during a musical performance, salivary volume increased and salivary sodium levels stabilised. The performance situation causes a rise in salivary potassium, which persists after beta blocking [48].

The anxiolytic effect of propranolol for performance anxiety [2,48,92] may be due to impaired retrieval of learned fear. Furthermore, propranolol injections into the dorsal periaqueductal grey (DPAG) have an anxiolytic effect because they increase the release of 5-HT, acting on post-synaptic 5-HT2 receptors, resultant from blockade of 5-HT1B autoreceptors that inhibit amine release from serotonergic nerve endings [136].

## 7. Mechanism of Action of Propranolol in the Treatment of PTSD

By decreasing retrieval of fear memories via the dorsal medial prefrontal cortex (dmPFC) and improving contextual safety learning via the HPC, beta-blockade can stop the recurrence of fear [137]. Furthermore, a reduction in diastolic BP was positively linked with a decrease in the severity of PTSD, confirming the anxiolytic effects of blockers via peripheral and central noradrenergic processes [138]. Stress-related NE release and compensatory downregulation β-adrenergic receptors (β-ARs) in the heart and peripheral vessels appear to play a role in these physiological effects [53]. The MAPK and JAK/STAT3 pathways may be among the biochemical pathways by which propranolol (and NE itself) influences aversive learning and memory processes, albeit this understanding is still in its early stages. According to Johansen, LeDoux, and colleagues, the MAPK pathway interacts with postsynaptic β-adrenergic signalling in the lateral nucleus of the amygdala (AMG) to modify the formation and consolidation of fear memories [139]. Other research has connected NE (and propranolol) with IL-6 signalling, and one group suggested that infusion of the inflammatory cytokine IL-6 into the basolateral amygdala (BLA) in rats may alter fear extinction learning, possibly through the JAK/STAT3 pathway [140,141].

The sympathetic nervous system’s excessive activity is a defining characteristic of PTSD [142,143,144,145]. The postulated therapeutic mechanism of action of propranolol is that beta-blockers prevent the binding of adrenaline and NE at the receptors (beta-1 and beta-2 for adrenaline, beta-1 for NE) [29]. The noradrenergic system is of key importance in modulating memory processes, and it has been found that stimulation of β-ARs facilitates the reconsolidation of emotional memory [146]. One of the promising strategies to treat PTSD is to pharmacologically block memory reconsolidation of the traumatic event [147]. Propranolol has been shown to interfere with memory reconsolidation [148]. Both memory formation and memory dissociation from emotional reaction may be inhibited by propranolol [29]. Propranolol selectively blocks protein synthesis, thereby prohibiting fear memory reconsolidation while leaving declarative memory unaffected [54]. This medicine crosses the blood–brain barrier and interferes with the neurobiological cyclic adenosine monophosphate (cAMP)/protein kinase A (PKA)/cAMP response element binding protein (CREB) cascade that is involved in the reconsolidation of destabilised fear memories by acting on β-ARs in the AMG, a brain area crucial for emotional regulation [139,149,150]. The increased release of NE, activation of β-AR, and higher surface expression of GluA1-containing α-amino-3-hydroxy-5-methyl-4-isoxazole propionic acid receptors (AMPARs), particularly the surface expression of extrasynaptic GluA1 in the lateral amygdala (LA), all contribute to the fear reactivation-induced preservation of fear memories. By altering the stability of GluA1 in LA, propranolol successfully prevented the changes brought on by NE and reactivation [151]. The AMG’s ability to activate and store memories is thought to be modulated by stress hormones, which propranolol is known to inhibit [152]. Propranolol can help with stress-related symptoms via affecting AMG-dependent memory reconsolidation and peripheral noradrenergic signalling [51]. Preclinical studies have shown that a single dose of propranolol administered immediately after an intense stressor (exposure to a predator/predator scent, electric shock) blocks subsequent expression of anxiety-related behaviour [153,154,155], possibly by impairing the consolidation of the stressful experience. Propranolol reaches its highest systemic concentration 75 min after intake [66]. Additionally, long-term use of propranolol may successfully lower tonically increased NE signalling in PTSD sufferers (i.e., those who would no longer benefit from acute propranolol paired with exposure therapy) [83].

Propranolol has been shown to reduce fear expression by modifying network-related activity and diminishing the reactivation of the initial traumatic memory trace [156]. Propranolol’s effects were demonstrated to be centrally mediated, with impaired fear memory retrieval that was context specific, contrary to an anxiolytic effect or alterations in generalised fear, according to behavioural controls [156]. The acute effects of propranolol are linked to: (1) decreased functional connectivity within and between the HPC, prefrontal cortex (PFC), and AMG regions; (2) decreased activity in the LA and infralimbic area (ILA); (3) changes in memory trace reactivation in the dorsal dentate gyrus (dDG) and BLA; and (4) changes in the correlation between memory trace reactivation in the anterior cingulate area (ACA), LA, and dorsal cornu ammonis 3 (dCA3) and freezing [156]. While enhanced dorsolateral PFC activity during the anticipation of unpleasant stimuli is connected with reduced symptom severity and better visuomotor PS in PTSD, greater AMG activation has been linked to slower visuomotor PS [156]. Despite propranolol’s ability to block access to the fear memory engram in the HPC during memory retrieval, it is probable that decreased activity in the ILA reduced extinction learning [22]. The dorsal HPC (dHPC) is essential for learning and memory associated with exploration and spatial navigation [157]. Propranolol’s acute effect on fear behaviour could be attributed to a decreased reactivation of the contextual components of fear memory in the dHPC [156]. The fear memory reactivation rate of encoding cells in the ACA was positively correlated with freezing levels in the control group, but not with propranolol, demonstrating that the ACA plays a role in fear memory encoding and retrieval, as well as mediating propranolol’s effects [156]. In addition, the ILA, which has fear-dampening/proextinction effects, becomes positively correlated with the ventral CA3 (vCA3) under propranolol [156]. The ventral HPC (vHPC) is thought to harbour a memory component of emotional valence, and, therefore, a greater inhibitory influence of the ILA on the vCA3 could influence fear retrieval. Disruption of the connection between these regions, which contain different components of the memory trace, may play a role in the behavioural effect [156].

## 8. Therapeutic Effects of Stage Fright Treatment with Propranolol in Comparison with Selected Drugs for This Indication

Although most of the works devoted to studying the effect of propranolol in the stage fright treatment are solely non-comparative placebo-controlled studies [158,159,160] (Table 1), in some cases, the efficacy of this API has been compared with other medications.

Brantigan et al. [48] considered the effectiveness of propranolol with terbutaline, a β_2_-adrenergic receptor agonist, in the treatment of stage fright on a group of musicians. It was a double-blind crossover study of the effects of the drugs given 1.5 h before recitals on 2 successive days. The performers preferred the use of propranolol in all categories: nervousness, anxiety, tremor, sweating, accuracy, style, ease of performance, control, tempo, rhythm, patience, memory, and comfort, and those results were statistically significant. Further, the application of propranolol successfully lowered both the average and maximum pulse during the performance.

Albus et al. [161] studied the autonomous reactions to different stressors and found that propranolol (40 mg) was more effective than diazepam (10 mg) and isamoltane (4 mg) in reducing excessive HR, elevated plasma NE levels, and BP. In another study, File and Lister [162] compared the effects of lorazepam with propranolol on experimentally-induced performance anxiety in a double-blind crossover study. While the high dose of lorazepam impaired performance and increased subjects’ ratings of dizziness, such changes were not observed among those receiving propranolol. Moreover, lorazepam but not propranolol increased ratings of sedation. Propranolol decreased and lorazepam increased subjects’ pulse.

The therapeutic benefit of combining propranolol with diazepam over either of these drugs given alone was tested by Hallstrom et al. [163] in a placebo-controlled crossover study with 24 patients. The addition of propranolol to treatment with diazepam significantly improved the outcome for psychic symptoms. A surprising finding of that study was that propranolol was ineffective in the treatment of somatic symptoms. Conversely, psychic symptoms were helped to a significant degree.

However, there are also reported studies in which propranolol was found to be less effective than the alternative drug. For example, Chaturvedi [164] showed that metoprolol, a selective β_1_ adrenoceptor antagonist, has been found to reduce anxiety symptoms more effectively and also seems to have fewer side effects as compared to propranolol. However, as the author admits, from that study no very conclusive statement could be made about the comparative efficacy of metoprolol over propranolol because of a limited number of subjects, as well as the method being non-blind. The sample was also relatively small and the duration of treatment quite short to record any appreciable side effects. Ibrahim and Atallah [165] conducted a comparative prospective double blind randomised study to test ivabradine versus propranolol given orally in microlaryngoscopic surgeries for attenuating stress response. The authors concluded that ivabradine was more effective than propranolol, with less changes in BP and HR values.

**Table 1:** Studies evaluating effect of propranolol on stage fright (performance anxiety) symptoms.

Study	Study Design	Participants	Reconsolidation Procedure	Outcome Measures	Results
Brantigan et al., 1982 [48]	Double blind, crossover study, placebo controlled	29 subjects, no information provided about age or sex	Propranolol and placebo given 1.5 h before recitals on 2 successive days	Continuous telemetric monitoring of the electrocardiogram (EKG). BP was recorded at the time of initial physical examination, before and after each performance. Subjects were observed during the performance for outward signs of stage fright	Propranolol eliminates the physical impediments to performance caused by stage fright; by eliminating the physical impediments to performance, propranolol can increase the quality of musical performance
Brewer, 1972 [90]	Placebo controlled, single blind	Psychology students before examination. According to the researchers, the “objective” stress was kept fairly constant, and the subjects were unusually homogeneous in terms of age, intellectual ability, and social class	Subjects were given increasing doses of propranolol until their resting pulse was slowed to between 55 and 65 beats per minute. The dosage necessary for manifest P-blockade ranged from 10 mg to 80 mg, and each student in the trial took an individually “tailored” dose of propranolol, or placebo, before the examination	Pulse monitoring	The results show clearly that propranolol causes no impairment of examination performance, and they suggest that it may actually improve performance in those who would otherwise be handicapped by severe anxiety, especially if cardiovascular symptoms are prominent
Drew et al., 1985 [92]	Randomised, double-blind, placebo controlled	30 junior doctors aged 23–33 years	Each participant underwent 2 examinations; 4.5 h before each test, candidates were given either 120 mg of propranolol or a matching placebo	Scores from the exams testing powers of mental arithmetic and verbal reasoning	This study has shown that propranolol treatment is associated with a small, but statistically significant, improvement in performance of simple tests of verbal reasoning and mental arithmetic, conducted in an atmosphere of mild stress
Faigel, 1991 [49]	Uncontrolled open-label trial	Total 32 students, 11 girls and 21 boys, in their senior year of high school.	A single 40 mg tablet of propranolol one hour before the second exam	Scores from the two consecutive exams, including the Scholastic Aptitude Test (SAT)	The mean improvement in the SAT verbal score was 50 points (95% confidence interval 30 to 60, *p* < 0.01). The mean increase in the math score was 80 points (95% confidence interval 60 to 90, *p* < 0.01). Propranolol may be effective in disabling performance anxiety associated with taking the exam test
Giddens et al., 2010 [158]	Double-blind, placebo controlled	12 adults, 6 male and 6 female, between the ages of 20 and 29 years	Each participant was given either 40 mg propranolol or placebo, as dictated by the randomisation schedule, with a glass of water. A delay of 60–75 min to allow for propranolol absorption occurred between treatment administration and the examination.	The intent of this experiment was to examine the effects of stress and beta-blockade on the voice. The parameters of F_0_, voice onset time (VOT), speaking rate, jitter, shimmer, maximum airflow declination rate, and subglottal pressure were measured under laboratory-induced sympathetic activation and beta-adrenergic blockade	Blockade of the increase in speaking rate in the propranolol treatment group may have indicated a reduction in cold pressor-induced anxiety; vocal jitter was observed to increase during beta-adrenergic blockade and stress
Stone et al., 1973 [91]	Double-blind, placebo controlled	24 college men ages 21 to 28	60 mg of propranolol hydrochloride in 6 divided doses (each of them 10 mg) given orally during the 12 h preceding experimental procedures	Plasma free fatty acid (FFA) concentration, anxiety measured from the verbal samples using the method of Gottschalk and Gleser	The correlation between anxiety scored from the initial speech sample and FFA level was positive and significant for the placebo subjects and negative for the propranolol group. Propranolol administered orally may have value as an antianxiety agent and, in addition, is seen as providing an avenue for the exploration of psychobiological relationships
Elman et al., 1998 [166]	Randomised, double-blinded, crossover study	5 3rd year ophthalmology residents, male, under age 30. 73 surgical cases were performed by the residents; the surgeons (residents) were administered propranolol for 40 cases and placebo for 33	Participants ingested a capsule containing either propranolol, 40 mg, or placebo 1 h prior to performing ophthalmic microsurgery	At the conclusion of each case, both the resident and attending surgeon observer independently completed a form by grading on a sliding scale: (1) amount of overall tremor, (2) amount of tremor during placement of the first 3 sutures after delivery of the lens or lens nucleus (in accordance with the prevailing surgical technique at the time the study was performed), (3) anticipated difficulty of case, (4) actual difficulty of case, and (5) anxiety (surgeon only)	There was a highly significant effect of propranolol in decreasing anxiety (*p* = 0.0058), reducing surgical tremor overall (*p* < 0.0001), and reducing tremor while placing the first 3 sutures following lens extraction (*p* < 0.0001). Propranolol, 40 mg, administered 1 h prior to surgery, significantly decreases tremor and anxiety in the surgeon without untoward effects to the surgeon and the patient

## 9. Therapeutic Effects of PTSD Treatment with Propranolol in Comparison with Selected Drugs for this Indication

In an uncontrolled trial, paroxetine therapy improved verbal memory function in PTSD patients [167]. According to Fani et al. [168], paroxetine enhanced verbal declarative memory in PTSD patients, however, this improvement was not statistically different from placebo.

A combination of atenolol (selective beta blocking agent) and scopolamine (an antimuscarinic agent) has been suggested as a fast-acting anxiolytic for the treatment of acute anxiety, particularly before medical procedures (dental, minimally invasive) [169]. Atenolol may control symptoms in patients with anxiety or PTSD, according to recent findings from retrospective research, with reported efficacy of up to 86% at doses of 100 mg/day. Compared with propranolol, the main benefit is higher propranolol tolerance, but the drawback is that there are no randomised clinical trials on this topic yet [170].

According to one study, metoprolol lowered the risk of developing PTSD-like symptoms in women after cardiac surgery [171].

The exaggerated fear response associated with PTSD has been reduced by pharmacotherapies that dampen NE transmission, such as the α_1_-adrenoceptor antagonist prazosin and the α_2_ agonist clonidine [34,172,173,174,175]. In humans, clonidine and propranolol have been shown to alleviate some PTSD symptoms [176,177,178]. Clonidine, an α_2_-adrenergic receptor agonist, reduces sympathetic outflow by acting on presynaptic receptors in the central nervous system. Many of the behavioural and physiological characteristics elicited by the psychosocial stress model of PTSD were prevented by chronic clonidine administration (anxiety-related behaviour, enhanced startle reaction, tachycardia, and elevated BP) [179]. When given immediately after stress exposure [180], clonidine prevented stress-induced changes in behaviour, and it normalised such changes when given before behavioural testing [181]. Clonidine has also been demonstrated to successfully block the reconsolidation of auditory fear memory [182], suggesting that it could be useful in reducing the strength of traumatic memories in PTSD patients. Clonidine has been shown to alleviate nightmares and hyperarousal symptoms in patients with PTSD [172,176,183,184].

Mifepristone caused a reduction in the conditioned response (CR) post-reactivation but not non-reactivation, and this reduction experienced only minimal restoration. When administered along with propranolol, mifepristone did not have any effect. Mifepristone post-reactivation had no negative effects on short-term memory. When administered systemically, it prevents rats from consolidating cue-conditioned fear. Concurrent administration of propranolol prevents this effect. Post-reactivation mifepristone may be a promising PTSD treatment, but not necessarily in combination with propranolol [185].

However, in a double-blind, placebo-controlled trial involving 66 adults with PTSD and comorbid major depression, Roullet et al. [66] replicated the traumatic memory reactivation from the initial trial by Brunet et al. [65]. They found no differences between propranolol and placebo on the severity of PTSD symptoms [66].

### Animal Clinical Trials

A preferred animal model that reflects some of the mechanisms involved in PTSD is fear conditioning [186]. Propranolol has been found to reduce exaggerated fear in PTSD patients [65] and rodents [187,188,189]. Only when propranolol was given before a delayed context re-exposure did it reduce fear expression. After propranolol administration, fear memory traces were disrupted in the dDG and BLA in ArcCreER^T2^ x eYFP mice. Propranolol alters activity in the HPC, PFC, and AMG regions during fear retrieval, modifying activity levels, functional connectivity, and the reactivation rates of fear memory traces. Propranolol severely impairs contextual fear expression [156]. Santos et al. [156] discovered a comparable acute effect of propranolol during the first tone test in a cued fear retrieval test. Furthermore, animal studies indicated that disrupting protein synthesis or blocking the β-AR after retrieval of a US reduced the expression of fear caused by numerous CSs [190,191].

Social defeat (SD) rats were protected from anxiety and depression-like behaviours by propranolol and nadolol. Certain ‘β-blockers’ may help to reduce the negative psychological effects of traumatic events. In rats, propranolol and nadolol treatment mitigated SD-induced depression-like behaviour as well as social interaction, suggesting that β_2_-ARs may play a role in mediating the anxiety response [192].

## 10. Materials and Methods

### 10.1. Protocol and Registration

**Figure 3:**
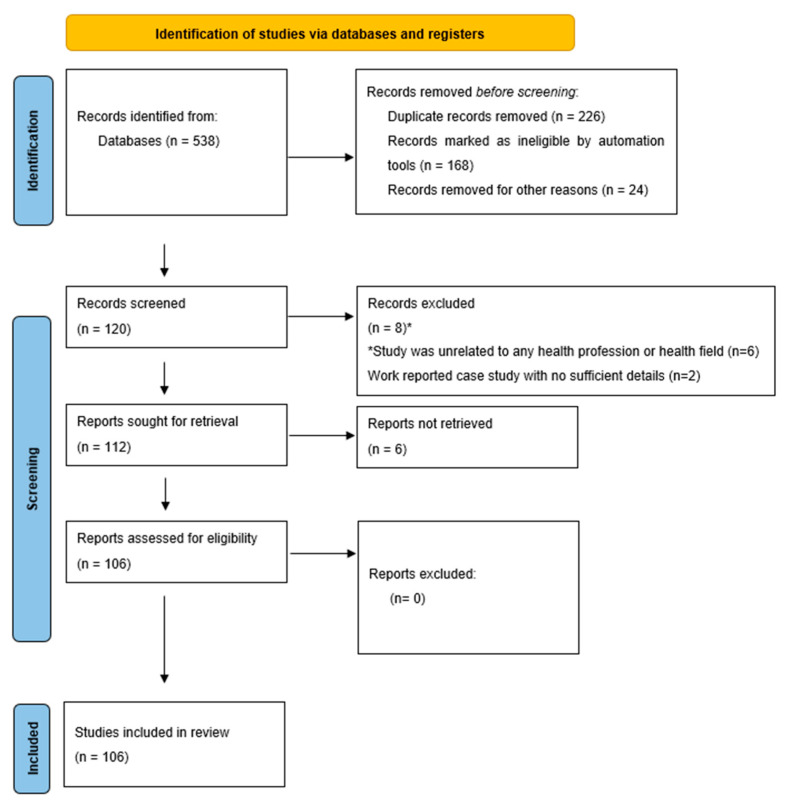
Flowchart based on the PRISMA statement.

The present review was performed according to the Preferred Reporting Items for Systematic Reviews (PRISMA) statement (Figure 3) [193], and it was registered in the Open Science Framework (OSF) (registration: https://doi.org/10.17605/OSF.IO/W9UNE, accessed 19 July 2022).

### 10.2. Research Question and Data Extraction

The research question was based on the PICO strategy for systematic exploratory reviews, where P = patients with PTSD or stage fright, I = application of propranolol, C = placebo or any other therapy, and O = reduction in PTSD or stage fright symptoms. The present study aimed to answer the following focused questions: “Is the administration of propranolol effective in the therapy of PTSD or stage fright”? Further data on the name of the first author, the date of publication, study design, dosage, outcome measures, and results were extracted from the articles included in this systematic review according to the eligibility criteria.

### 10.3. Study Design and Search Strategy

Two independent examiners (Ł.S. and D.F.) were chosen to select the articles. Thus, the examiners conducted a comprehensive literature search in the PubMed, Web of Science, Scopus, and Cochrane Library databases. The search terms were “propranolol”, “performance anxiety”, and “stage fright”. The search strategy was deliberately broad and based on a combination of keywords. Included articles were screened for additional relevant studies cited for inclusion in our analysis, if meeting the criteria. A manual search was also performed in other relevant journals in this field. Based on the titles and abstracts, the same two independent examiners selected and classified the articles as included in or excluded from the review. The Rayyan for Systematic Reviews software was used to delete duplicate articles [194]. The data were extracted from the articles selected after concluding the eligibility step. The studies were analysed and discussed. Any disagreement during the process was solved by reaching a consensus before proceeding to the next steps.

### 10.4. Study Selection and Criteria

In the screening process, two reviewers (Ł.S. and D.F.) independently screened all the imported publications in the Rayyan software. The inclusion criterion for this review was the use of propranolol in the treatment of PTSD or stage fright, including in combined therapy. There were no restrictions on study design (e.g., inclusion of in vitro and in vivo studies, observational human studies, and randomised clinical trials) and language. The exclusion criteria were review articles, case reports, letters, comments or other modalities, clinical trial protocols, or conference abstracts. Upon completion of inclusion and exclusion, any disagreements were resolved by consensus between the two reviewers.

## 11. Discussion

Despite its over half-century history, propranolol is still perceived as a valuable active pharmaceutical ingredient, being present, e.g., in the World Health Organization’s model list of essential medicines [195]. Surprisingly, its application on that list is not related to the cardiovascular effects that propranolol is most famous for, but for a migraine prophylaxis.

As shown in this review, there are well documented molecular bases for the application of propranolol in the treatment of various types of anxiety or stress, including stage fright and post-traumatic stress disorder. Due to its high lipophilicity and ability to cross the blood–brain barrier, propranolol exerts effects in the central nervous system in addition to its peripheral activity. Propranolol, being a pleiotropic agent, can surely be called a multi-target drug. This is why it can be possibly used to treat such complex conditions, that manifest themselves in both mental and somatic effects.

It is not uncommon for speakers and singers to use propranolol, unprescribed and prescribed, to combat the symptoms of stage fright. Investigators have reported a large range of changes in performance following beta-blockade. Performance quality has been reported to improve and the degree of pre-performance anxiety has been determined to decrease upon treatment with propranolol. Although beta-blockers are considered safe, contraindications to use and side effects, such as bronchospastic effects, bradycardia, and hypotension, do exist.

No studies of the effect of beta-blockage on stage fright or test anxiety have used formal objective tests to measure response to treatment. Instead, researchers have relied on the subjective scores given by raters of musical performances or on unstandardized end-of-term examinations in college students to demonstrate the effect of medication.

This review shows a lack of well-designed clinical studies in this topic. This limits the scientific evidence, allowing neither firm conclusions in favour or against the use of propranolol in the treatment of stage fright, nor recommendations for informed decision-making in clinical practice. No random clinical trials were available on the effects of propranolol in the treatment of stage fright.

With regard to the therapeutic effects of propranolol, it has been proposed that propranolol’s anxiolytic properties may result from its peripheral (autonomic) rather than its central activity. This may explain the lack of evidence for propranolol’s efficacy in the long-term treatment of anxiety disorders other than panic disorder. The present review was limited by the moderate number of small studies examining the effects of propranolol on anxiety disorders and by the risk of bias these trials presented. As withdrawal reasons were seldom reported, the possibility of selective loss to follow-up in some studies could not be ruled out.

In conclusion, the quality of evidence for the efficacy of propranolol at present is insufficient to support the routine use of propranolol in the treatment of stage fright, while it may be very beneficial in the therapy of PTSD.

## Data Availability

Not applicable.

## Abbreviations

The following abbreviations are used in this manuscript:

ACA	anterior cingulate area
AMG	amygdala
AMPAR	α-amino-3-hydroxy-5-methyl-4-isoxazole propionic acid receptor
API	Active Pharmaceutical Ingredient
ASD	Autism Spectrum Disorder
ATC	Anatomical Therapeutic Chemical Classification System
β-AR	beta-adrenergic receptor
BDI-II	Beck Depression Inventory-II
BLA	basolateral amygdala
BP	blood pressure
cAMP	cyclic adenosine monophosphate
CNS	central nervous system
CR	conditioned response
CREB	cAMP response element-binding protein
CS	conditioned stimulus
CSF	cerebrospinal fluid
dCA3	dorsal cornu ammonis 3
dDG	dorsal dentate gyrus
dHPC	dorsal hippocampus
dmPFC	dorsal medial prefrontal cortex
DPAG	dorsal periaqueductal grey
EBAD	emotional, behavioural, and autonomic dysregulation
EKG	electrocardiogram
FDA	Food and Drug Administration
FFA	free fatty acid
HPC	hippocampus
HR	heart rate
ILA	infralimbic area
LA	lateral amygdala
NE	noradrenaline, norepinephrine
OSF	Open Science Framework
PCL-S	PTSD Check List
PFC	prefrontal cortex
PKA	protein kinase A
PO	Perceptual Organisation
PRISMA	Preferred Reporting Items for Systematic Reviews
PS	Processing Speed
PTSD	post-traumatic stress disorder
SAD	social anxiety disorder
SAT	Scholastic Aptitude Test
SD	social defeat
SIB	self-injurious behaviour
SVTs	supraventricular tachyarrhythmias
US	unconditioned stimulus
vCA3	ventral cornu ammonis 3
vHPC	ventral hippocampus
VOT	voice onset time
WM	Working Memory
